# Supplementary material for: Large‐Scale Mapping of Moiré Superlattices by Hyperspectral Raman Imaging
Source: Adv Mater. 2021 Jul 9;33(34):2008333. doi: 10.1002/adma.202008333 (PMC11469034; doi:10.1002/adma.202008333)
Supplement: Supplementary file 1 — Supporting Information [file ADMA-33-2008333-s001.pdf]

© 2021 Wiley-VCH GmbH

ADVANCED MATERIALS

Supporting Information

for *Adv. Mater.,* DOI: 10.1002/adma.202008333

Large-Scale Mapping of Moiré Superlattices by Hyperspectral Raman Imaging

Kai-Qiang Lin, Johannes Holler, Jonas M. Bauer, Philipp Parzefall, Marten Scheuck, Bo Peng, Tobias Korn, Sebastian Bange, John M. Lupton, and Christian Schüller**

Supporting Information

Large-scale mapping of moiré superlattices by hyperspectral Raman imaging

Kai-Qiang Lin, Johannes Holler, Jonas M. Bauer, Philipp Parzefall, Marten Scheuck, Bo Peng, Tobias Korn, Sebastian Bange, John M. Lupton, Christian Schüller**

Supplementary Note 1. Twist-angle dependence of the moiré-phonon frequency

We calculate the twist-angle dependence of moiré-phonon frequency following the methodology detailed by Lin *et al.*[1] Briefly, we first obtain the phonon dispersion along the moiré reciprocal lattice direction by interpolating the phonon dispersion of monolayer WSe_2 along the Γ-M and Γ-K directions as

$$E_{\vec{g}}(k) = (\theta/60°) * E_{\overrightarrow{\Gamma \mathrm{M}}}(k) + (1 - (\theta/60°)) * E_{\overrightarrow{\Gamma \mathrm{K}}}(k), \#(1)$$

where the effective twist angle θ ranges between 0° and 30°, E is the phonon energy and k the momentum. Within the hexagonal lattice, the momentum provided by the moiré superlattice is related to the moiré period λ in real space by

$$k = 4\pi/(\lambda\sqrt{3}), \#(2)$$

and the moiré period is related to the twist angle through

$$\lambda = 0.5\ a/\sin(\theta/2), \#(3)$$

where a is the in-plane lattice constant and is set to 0.3282 nm according to the WSe_2 crystal structure[2]. With Equations (1-3), we can obtain the twist angle dependence of moiré phonon frequency $E_{\vec{g}}(\theta)$ as shown in Figure 2b in the main text.

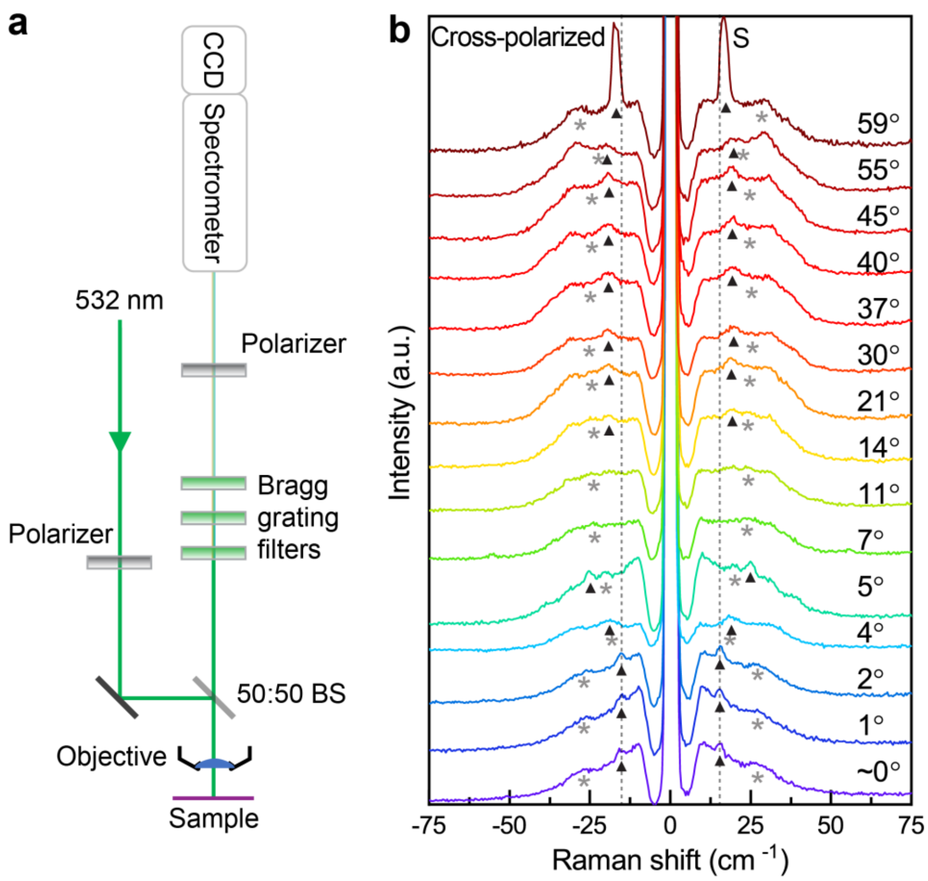

Figure S1. Setup configuration and cross-polarized low-frequency Raman measurements. a) Schematic configuration of the setup for low-frequency Raman scattering measurements. For unpolarized measurements described in the main text, the polarizer in the detection beam path is removed. For the cross-polarization measurement shown in panel b, the polarizer in the detection beam path is set orthogonal to the polarizer in the laser excitation beam path. b) Low-frequency Raman spectra of twisted bilayer WSe_2 measured in the cross-polarization configuration, where the interlayer breathing mode (*) is suppressed and the shear mode (S) is more prominent. The characteristic peaks which are less suppressed by the cross-polarization are marked by black triangles. The interlayer breathing mode is largely suppressed in the cross-polarization configuration. We note that the polarization selection rules of the interlayer breathing mode and the shear mode have only been validated in natural multilayers with well-defined symmetry. For bilayers with twist angles below 3°, the shear mode remains at a frequency of 15.3 cm^{-1}, independent of twist angle and thus indicative of an atomic reconstruction. With moiré phonons emerging in 4° and 5° twisted bilayers, the mode appears to shift to a higher frequency with

increasing twist angle, even though the shear mode is generally not expected to arise in twisted bilayers due to the periodicity mismatch between adjacent layers[3].

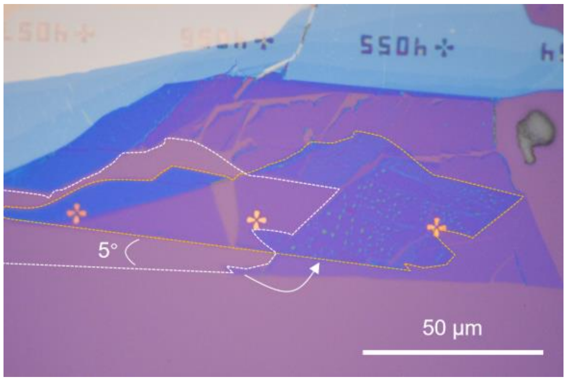

Figure S2. Illustration of twisted bilayer fabrication through sequential stamping. Optical microscopy image of the 5° twisted bilayer WSe_2 sample. The monolayer WSe_2 was first partially stamped onto the silicon chip and, after rotating the silicon chip by 5°, the remaining section (marked by dashed lines) of the monolayer was stamped on top of the first section.

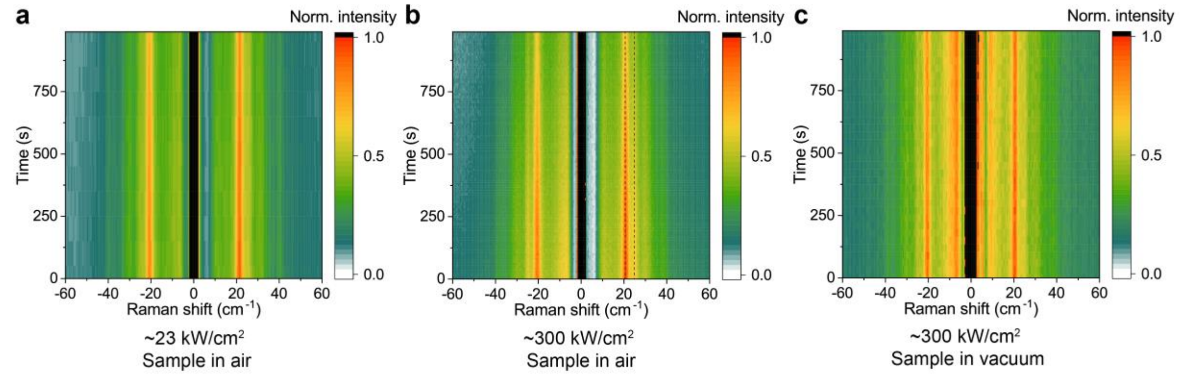

Figure S3. Time series of Raman measurements on the 5° twisted bilayer WSe_2 sample under different conditions of (a) ~23 kW/cm^2 irradiance and sample being in air, (b) ~300 kW/cm^2 irradiance and sample being in air, and (c) ~300 kW/cm^2 irradiance and sample being in vacuum.

Supplementary References

[1] M.-L. Lin, Q.-H. Tan, J.-B. Wu, X.-S. Chen, J.-H. Wang, Y.-H. Pan, X. Zhang, X. Cong, J. Zhang, W. Ji, P.-A. Hu, K.-H. Liu, P.-H. Tan, *ACS Nano* **2018**, *12*, 8770.

[2] W. J. Schutte, J. L. De Boer, F. Jellinek, *J. Solid State Chem.* **1987**, *70*, 207.

[3] a) J.-B. Wu, Z.-X. Hu, X. Zhang, W.-P. Han, Y. Lu, W. Shi, X.-F. Qiao, M. Ijiäs, S. Milana, W. Ji, A. C. Ferrari, P.-H. Tan, *ACS Nano* **2015**, *9*, 7440; b) J. Holler, S. Meier, M. Kempf, P. Nagler, K. Watanabe, T. Taniguchi, T. Korn, C. Schüller, *Appl. Phys. Lett.* **2020**, *117*, 013104.
